# Determination of cardiovascular risk in Spanish veterinarians using different scaling methods

**DOI:** 10.47626/1679-4435-2023-826

**Published:** 2023-04-18

**Authors:** Ángel Arturo López-González, Hilda María González San Miguel, Sebastiana Arroyo Bote, Pere Riutord Sbert, María del Mar Rigo Vives, José Ignacio Ramírez Manent

**Affiliations:** 1 University School ADEMA Palma, Balearic Islands, Spain; 2 Balearic Islands Health Service, Balearic Islands, Spain

**Keywords:** cardiovascular disease, hypertension, metabolic syndrome, non-alcoholic fatty liver disease, obesity, veterinarians, doenças cardiovasculares, obesidade, risco, médicos veterinários

## Abstract

**Introduction:**

Cardiovascular diseases are responsible for a significant morbimortality rate around the world. Due to the characteristics of their work, health care professionals, including veterinarians, are more prone to present this type of pathology.

**Objectives:**

To determine the level of cardiovascular risk using different scales in a group of veterinarians.

**Methods:**

A descriptive and cross-sectional study of 610 Spanish veterinarians was conducted to assess cardiovascular risk scores, including 14 overweight and obesity scales, six fatty liver scales, six cardiovascular risk scales, four atherogenic indices, and three metabolic syndrome scales.

**Results:**

The prevalence of obesity among women was 7.95%, and 17.53% among men. Hypertension was present in 15.23% of women and 24.68% of men. Dyslipidemia affected 45% of women and 58.64% of men. The prevalence of metabolic syndrome according to the International Diabetes Federation criteria was slightly over 10% while 10.90% of women and 14.93% of men showed moderate to high values on the Registre Gironí del Cor scale.

**Conclusions:**

There is a moderate to high level of cardiovascular risk among veterinarians in this group.

## Abstract (pt)

**Introdução:**

As doenças cardiovasculares são responsáveis por uma taxa de morbimortalidade significativa no mundo. Devido às características do seu trabalho, os profissionais de saúde, incluindo veterinários, são mais propensos a apresentar este tipo de patologia.

**Objetivos:**

Determinar o nível de risco cardiovascular utilizando diferentes escalas num grupo veterinários.

**Métodos:**

Estudo descritivo e transversal em 610 veterinários em que foram avaliadas diferentes escalas relacionadas com o risco cardiovascular, incluindo 14 escalas de excesso de peso e obesidade, seis de fígado gordo, seis de risco cardiovascular, quatro índices aterogênicos e três de síndrome metabólica.

**Resultados:**

A prevalência da obesidade foi de 7,95% nas mulheres e 17,53% nos homens. A hipertensão estava presente em 15,23% das mulheres e 24,68% dos homens. A dislipidemia afetou 45% das mulheres e 58,64% dos homens. A prevalência da síndrome metabólica com critérios da International Diabetes Federation foi ligeiramente superior a 10% enquanto valores moderados ou elevados da escala Registre Gironí del Cor foram encontrados em 10,90% das mulheres e 14,93% dos homens.

**Conclusões:**

Neste grupo de veterinários, o nível de risco cardiovascular pode ser considerado de moderado a elevado.

## INTRODUCTION

Cardiovascular diseases are currently considered to be the greatest cause of morbidity and mortality worldwide. According to data from the World Health Organization (WHO), each year more people die from this disease than from any other pathology.^1^ For this reason, this organization prepared an action plan aimed at reducing premature deaths associated with this cause, which focused especially on strengthening public services and policies that could have a particular impact on noncommunicable diseases, among which cardiovascular diseases play a very prominent role.^2^

There are many scales for assessing cardiovascular risk (CVR), some of which quantify it directly by determining the risk of suffering a cardiovascular event over a time period, usually 10 years, as is the case, for example, of the Registre Gironí del Cor (REGICOR),^3^ the Dyslipidemia Obesity and Cardiovascular Risk in Spain (DORICA),^4^ or the Spanish Cardiovascular Risk Equation (ERICE)^5^ in Spain. Other scales quantify the risk of suffering a fatal cardiovascular event over a time period, as is the case with the Systematic Coronary Risk Evaluation (SCORE) scale.^6^ In recent years, other direct risk scales have been developed based on the aforementioned scales, such as the heart age and vascular age scales. In addition to the scales that assess direct CVR, there are others that do so indirectly, such as scales for overweight and obesity, atherogenic indices, metabolic syndrome, or fatty liver disease. All these scales will be discussed more extensively in the methodology section.

Many studies have determined CVR in both the general and the working population^7^ but to the best of our knowledge, no study has been conducted with veterinarians. As a result of their work in the care of suffering animals, many of these professionals are at risk of compassion fatigue and occupational stress,^8^ which is an important trigger for cardiovascular disease.

The objective of this study was to determine the level of CVR in Spanish veterinarians and the influence of age, sex, and tobacco consumption on this risk.

## METHODS

A descriptive, cross-sectional study was performed on 649 Spanish veterinarians between January 2019 and December 2019. Thirty-nine of them were excluded (3 for not agreeing to participate, 13 for a history of previous cardiovascular events, and 23 for lack of the parameters necessary to calculate the different CVR scales), thus 610 veterinarians remained. A total of 302 (49.51%) were women (mean age 42.51 years), and 308 (50.49%) were men (mean age 43.35 years).

Participants were selected among workers who attended periodic occupational medical checkups in companies in different regions of Spain.

### INCLUSION CRITERIA

- Consent to participate in the study and to use of the data for epidemiological purposes.
- No previous cardiovascular events.

Following standardization of measurement techniques, healthcare professionals from the different occupational health units that participated in the study conducted anthropometric, clinical, and analytical assessments.

The following parameters were included in the assessment:

- Weight (in kg) and height (in cm) were determined with a SECA 700 scale and a SECA 220 measuring rod.
- Abdominal waist circumference (in cm) was measured with a SECA 200 tape measure. For the waist-to-height ratio, the cut-off point was set at 0.50.
- Blood pressure was measured in the supine position with a calibrated OMRON M3 automatic sphygmomanometer and after a 10-minute rest period. Three determinations were made at 1-minute intervals, obtaining the mean of the three. Hypertension was considered when the values were equal to/higher than 140 mmHg systolic or 90 mmHg diastolic blood pressures or if the worker was receiving antihypertensive treatment.

Blood glucose, total cholesterol and triglycerides were determined by peripheral venipuncture after fasting for at least 12 hours. Glycemia, total cholesterol and triglycerides were determined by automated enzymatic methods. HDL was determined by precipitation with dextran sulfate Cl2Mg and LDL was calculated using the Friedewald formula (provided that triglycerides were less than 400 mg/dL). All the above values are expressed in mg/dL. Friedewald’ formula:


LDL-c=total cholesterol -HDL-c- triglycerides/5

The following were considered abnormal values: 200 mg/dL for cholesterol, 130 mg/dL for LDL, and 150 mg/dL for triglycerides or if they were under treatment for any of these factors.

Blood glucose values were classified according to the criteria of the American Diabetes Association (ADA) and are considered to be diabetes at 126 mg/dL or if individuals are receiving hypoglycemic treatment.

Body mass index (BMI) was calculated by dividing weight by height in meters squared. Obesity was considered to be 30 kg/m^2^ or more.

We used the following scales to estimate overweight and obesity: Clínica Universitaria de Navarra Body Adiposity Estimator (CUN BAE),^9^ Equation Córdoba Estimator Body Fat (ECORE-BF),^10^ Palafolls’s formula,^11^ Deurenberg’s formula,^12^ relative fat mass,^13^ visceral adiposity index (VAI),^14^ dysfunctional adiposity index,^15^ body roundness index,^16^ body surface index (BSI),^17^conicity index,^18^ body shape index (ABSI),^19^ and normalized weight-adjusted index (NWAI).^20^ To measure insulin resistance we used the following indices: triglyceride glucose index, triglyceride glucose index-BMI, triglyceride glucose index-waist.^21^ For cardiometabolic risk: waist triglyceride index,^22^ cardiometabolic index,^23^ fatty liver index,^24^ hepatic steatosis index (HSI),^25^ Zhejian University index (ZJU),^26^ fatty liver disease index (FLD),^27^ BARD scoring system,^28^ Framingham steatosis index,^29^ and lipid accumulation product.^30^ The different scales are shown in Table 1.

**Table 1:** Different scales used in the study

Determination	Scale	Parameters
Overweight-obesity	CUN BAE	Age, gender, BMI
	ECORE-BF	Age, gender, BMI
	Palafolls’s formula	Gender, BMI, waist
	Deurenberg’s formula	Age, gender, BMI
	Relative fat mass	Gender, height, waist
	VAI	Gender, BMI, waist, HDL, triglycerides
	Dysfunctional adiposity index	Gender, BMI, waist, HDL, triglycerides
	Body roundness index	Waist, height
	BSI	Weight, height
	Conicity index	Weight, height, waist
	ABSI	Waist, height, BMI
	NWAI	Weight, height
Insulin resistance	Triglyceride glucose index	Glucose, triglycerides
Cardiometabolic risk	Waist triglyceride index	Waist, triglycerides
	Cardiometabolic index	Height, waist, HDL, triglycerides
Fat liver	Fatty liver index	Triglycerides, BMI, GGT, waist
	Hepatic steatosis index	AST, ALT, BMI, diabetes, gender
	Zhejiang University index	Triglycerides, BMI, glucose, ALT, AST, gender
	Fatty liver disease index	Triglycerides, BMI, glucose, ALT, AST
	BARD scoring system	AST, ALT, BMI, diabetes
	Framingham steatosis index	Age, gender, BMI, triglycerides, hypertension, diabetes, AST, ALT
	Lipid accumulation product	Waist, triglycerides, gender

ABSI = body shape index; BMI = body mass index; BSI = body surface index; CUN BAE = Clínica Universitaria de Navarra Body Adiposity Estimator; ECORE-BF = Equation Córdoba Estimator Body Fat; NWAI = normalized weight-adjusted index; VAI = visceral adiposity index.

The atherogenic indexes determined were:

- Total cholesterol/HDL (considered as high > 5 in men and > 4.5 in women), LDL/HDL and triglycerides/HDL (high > 3);
- Triglycerides/HDL (high >3);
- Total cholesterol-HDL (high >130).
- Cardiometabolic indicators:
- Hypertriglyceridemic Waist Phenotype;^31^
- Waist > 102 cm (men) > 88 cm (women); and
- Triglycerides > 150 mg/dL or treatment of hypertriglyceridemia.

Metabolic syndrome was determined using three models:^32^

- National Cholesterol Educational Program Adult Treatment Panel III (NCEP ATP III), which considers metabolic syndrome when three or more of the following factors are present: waist circumference is greater than 88 cm in women and 102 in men; triglycerides > 150 mg/dL or specific treatment for this lipid disorder; blood pressure > 130/85 mmHg; HDL < 40 mg/dL in women or < 50 mg/dL in men or specific treatment is followed, and fasting blood glucose > 100 mg/dL or specific glycemic treatment.
- The International Diabetes Federation (IDF) model, which considers the presence of central obesity necessary, defined as a waist circumference of > 80 cm in women and > 94 cm in men, in addition to two of the other factors mentioned above for ATP III (triglycerides, HDL, blood pressure and glycemia).
- The JIS model, which follows the same criteria as NCEP ATPIII but the waist circumference cut-off points start at 80 cm in women and 94 cm in men.

Atherogenic dyslipidemia^33^ is characterized by high triglyceride concentrations (> 150 mg/dL), low HDL (< 40 mg/dL in men and < 50 mg/dL in women), and normal or slightly high LDL. If LDL values are high (> 160 mg/dL) we speak of lipid triad.

### CVR SCALES USED

REGICOR is an adaptation of the Framingham scale for the Spanish population^3^ and assesses the risk of suffering a cardiovascular event over a 10-year period. It can be applied between 35 and 74 years of age. The risk is considered moderate at 5% or above and high at 10% or above.^6^

The SCORE scale for low-risk countries is used in Spain^6^ to determine the risk of suffering a fatal cerebrovascular event over a 10-year period. It can be calculated between 40 and 65 years of age. Moderate risk is defined at 4% and high risk at 5% or above.

DORICA. This scale is based on the DORICA study,^4^ which was conducted in a very large Spanish population base. It estimates the risk of suffering a fatal or non-fatal cerebrovascular event over a 10-year period. The tables are applied to people between 25 and 64 years of age. To calculate risk one assesses age, sex, smoking, diabetes, systolic and diastolic blood pressure, total cholesterol, and HDL-c. To classify the level of risk with the DORICA tables, the cut-off points recommended by the authors determine that risk is moderate when it is between 10 and 19%, high from 20%, and very high if it exceeds 39%.

ERICE is based on seven population-based cohort studies conducted in different geographical areas of Spain.^5^ It estimates the risk of suffering a fatal or non-fatal cerebrovascular event over a 10-year period. The tables apply to individuals between 30 and 80 years of age. To calculate risk, age, sex, smoking, diabetes, systolic blood pressure, antihypertensive treatment, and total cholesterol are taken into account. To classify the level of risk with the ERICE tables, the cut-off points recommended by the group responsible for the study were used: risk is considered moderate if it exceeds 5%, moderate-high if it is between 15 and 19%, high if it is between 20 and 39%, and very high if it exceeds 39%.

To calculate vascular age with the Framingham model^34^ we need age, sex, HDL-c, total cholesterol, systolic blood pressure values, antihypertensive treatment, smoking, and diabetes. It can be calculated from the age of 30 years.

In turn, to calculate vascular age with the SCORE model^35^ the variables analyzed were age, sex, systolic blood pressure, smoking and total cholesterol. As with the scale from which it is derived, it can be calculated in individuals between 40 and 65 years of age.

An interesting concept applicable to both vascular ages is avoidable lost life years (ALLY), which can be defined as the difference between biological age and vascular age.

A smoker is considered to be any person who has regularly consumed at least one cigarette/day (or the equivalent in other types of consumption) in the last month, or has quit smoking less than 12 months ago.

### STATISTICAL ANALYSIS

A descriptive analysis of the categorical variables was conducted, calculating the frequency and distribution of responses for each of them. For quantitative variables, the mean and standard deviation were calculated, and for qualitative variables, the percentage was calculated. The bivariate association analysis was performed using the X^2^ test (with correction of Fisher’s exact statistic when conditions required) and Student’s *t* test for independent samples. For multivariate analysis, binary logistic regression was used with the Wald method, with calculation of the odds ratio and the Hosmer-Lemeshow goodness-of-fit test. Statistical analysis was performed with SPSS version 27.0, and statistical significance was set at 0.05.

### ETHICAL ASPECTS

The study was approved by the Research Ethics Committee of the Illes Balears health area no. IB 4383/20. All procedures were performed in accordance with the ethical standards of the institutional research committee and with the 2013 Declaration of Helsinki. All patients signed written informed consent documents prior to participation in the study.

## RESULTS

Clinical variables show statistically higher values in men, except for total cholesterol, LDL cholesterol, and AST. Smoking prevalence is high, especially among women. The complete data are presented in Table 2.

**Table 2:** Characteristics of the veterinarians

	Women (n = 302)	Men (n = 308)	p-value
	Mean (SD)	Mean (SD)	
Age (years)	42.51 (9.97)	43.35 (8.79)	0.269
Height (cm)	160.48 (6.26)	175.98 (6.18)	< 0.0001
Weight (cm)	60.92 (11.99)	77.13 (10.43)	< 0.0001
Waist (cm)	71.78 (7.92)	84.22 (9.44)	< 0.0001
Systolic blood pressure (mmHg)	116.56 (17.24)	126.18 (12.97)	< 0.0001
Diastolic blood pressure (mmHg)	71.56 (9.71)	78.29 (9.90)	< 0.0001
Total cholesterol (mg/dL)	211.05 (50.08)	206.05 (35.98)	0.157
HDL-c (mg/dL)	61.48 (14.83)	50.70 (8.55)	< 0.0001
LDL-c (mg/dL)	133.06 (44.50)	132.90 (36.74)	0.961
Triglycerides (mg/dL)	82.68 (37.85)	112.19 (59.31)	< 0.0001
Glycaemia (mg/dL)	84.97 (12.15)	93.81 (13.09)	< 0.0001
ALT (U/l)	21.20 (8.29)	25.52 (14.81)	< 0.0001
AST (U/l)	20.75 (5.72)	19.40 (6.21)	0.507
GGT (U/l)	21.81 (9.54)	26.86 (16.95)	< 0.0001
	Percentage	Percentage	p-value
< 40 years	41.72	35.07	0.036
40-49 years	29.14	38.96	
≥ 50 years	29.14	25.97	
Non-smokers	56.95	68.83	0.020
Smokers	43.05	31.17	

SD = standard deviation.

In this study, men showed higher mean values on all scales except those measuring body fat percentage, which are higher in women. The rest of the scales analyzed were overweight and obesity, metabolic syndrome, fatty liver, and CVR scales as well as atherogenic indices that showed higher mean values in men. The data are presented in Table 3.

**Table 3:** Mean values of the different cardiovascular risk scales in veterinarians according to sex

	Women (n = 302)	Men (n = 308)	p-value
	Mean (SD)	Mean (SD)	
Waist-to-height ratio	0.45 (0.05)	0.48 (0.05)	< 0.01
BMI	23.70 (4.83)	24.91 (3.18)	< 0.01
CUN BAE	33.33 (7.15)	23.50 (4.97)	< 0.01
ECORE-BF	33.15 (7.51)	23.56 (4.94)	< 0.01
Relative fat mass	30.78 (4.79)	21.73 (4.48)	< 0.01
Palafolls’s formula	36.99 (5.26)	27.88 (3.39)	< 0.01
Deurenberg’s formula	32.81 (6.95)	23.66 (4.87)	< 0.01
BSI	47.47 (7.31)	55.37 (5.67)	< 0.01
NWAI	0.04 (1.26)	0.11 (0.99)	< 0.01
Body roundness index	2.47 (0.86)	3.02 (0.94)	< 0.01
ABSI	0.07 (0.01)	0.08 (0.01)	< 0.01
VAI	2.33 (1.27)	6.29 (3.95)	< 0.01
Dysfunctional adiposity index	0.60 (0.32)	0.82 (0.48)	< 0.01
Conicity index	1.08 (0.09)	1.17 (0.10)	< 0.01
Fatty liver index	16.31 (19.70)	24.01 (22.46)	< 0.01
Hepatic steatosis index	30.55 (3.57)	39.44 (7.81)	< 0.01
Zhejiang University index	32.09 (1.72)	39.24 (5.25)	< 0.01
Fatty liver disease index	25.43 (1.98)	34.06 (5.25)	< 0.01
Lipid accumulation product	13.53 (11.38)	25.36 (18.67)	< 0.01
BARD scoring system	0.68 (0.79)	0.95 (1.07)	< 0.01
Framingham steatosis index	0.05 (0.02)	0.32 (0.18)	< 0.01
Triglyceride glucose index	8.07 (0.43)	8.44 (0.55)	< 0.01
Triglyceride glucose index-BMI	192.27 (46.05)	210.76 (34.03)	< 0.01
Triglyceride glucose index-waist	579.80 (76.68)	711.66 (100.22)	< 0.01
Triglyceride glucose index-WtHR	3.62 (0.49)	4.04 (0.54)	< 0.01
Waist triglyceride index	67.72 (33.95)	107.76 (58.91)	< 0.01
ALLY vascular age SCORE^*^	5.98 (6.64)	6.19 (3.90)	0.72
SCORE scale^*^	0.36 (0.83)	0.98 (0.90)	< 0.0021
ALLY vascular age Framingham†	2.61 (13.65)	5.17 (8.23)	0.01
REGICOR scale‡	2.19 (1.76)	2.81 (1.95)	< 0.0011
ERICE scale†	3.24 (3.35)	3.62 (3.16)	0.17
DORICA scale	2.78 (3.56)	5.28 (3.50)	< 0.01
Nº factors of metabolic syndrome NCEP ATPIII	0.64 (0.79)	1.23 (1.11)	< 0.01
Nº factors of metabolic syndrome JIS	0.80 (1.01)	1.64 (1.23)	< 0.01
Cardiometabolic index	0.65 (0.43)	1.12 (0.70)	< 0.01
Atherogenic index total cholesterol/HDL-c	3.56 (1.02)	4.21 (1.23)	< 0.01
Atherogenic index triglycerides/HDL-c	1.42 (0.83)	2.30 (1.36)	< 0.01
Atherogenic index LDL-c/HDL-c	2.28 (0.91)	2.75 (1.10)	< 0.01
Atherogenic index total cholesterol-HDL-c	149.56 (48.52)	155.36 (37.61)	0.1

* Women n = 176, men n = 192.

† Women n = 268, men n = 284.

‡ Women n = 248, men n = 268.

ABSI = body shape index; ALLY = avoidable lost life years; BMI = body mass index; BSI = body surface index; CUN BAE = Clínica Universitaria de Navarra Body Adiposity Estimator; DORICA = Dyslipidemia Obesity and Cardiovascular Risk in Spain; ECORE-BF = Equation Córdoba Estimator Body Fat; ERICE = Spanish Cardiovascular Risk Equation; NCEP ATPIII = National Cholesterol Educational Program Adult Treatment Panel III; NWAI = normalized weight-adjusted index; REGICOR = Registre Gironí del Cor; SCORE = Systematic Coronary Risk Evaluation; SD = standard deviation; VAI = visceral adiposity index.

According to our analysis of the prevalence of high scores on a variety of CVR- related scales, in most cases this values were higher among men, while the most unfavorable values were found only in women with some scales that assess body fat, such as CUN BAE, ECORE-BF, Relative Fat Mass and Deurenberg’s formula, metabolic syndrome with the IDF criteria, Lipid triad, and atherogenic index total cholesterol/HDL. The complete data can be found in Table 4.

**Table 4:** Prevalence of altered values of the different cardiovascular risk scales by gender in veterinarians

	Women (n = 302)	Men (n = 308)	p-value
	Percentage	Percentage	
Waist-to-height ratio > 0.50	19.87	36.36	< 0.01
BMI obesity (≥ 30kg/m^2^)	7.95	17.53	< 0.01
CUN BAE obesity (> 35 women, > 25 men)	37.09	27.92	0.02
ECORE-BF obesity (> 35 women, > 25 men)	36.42	27.92	0.00
Relative fat mass obesity (> 35 women, > 25 men)	45.03	27.27	< 0.01
Palafolls’s formula obesity (> 35 women, > 25 men)	59.60	76.62	< 0.01
Deurenberg’s formula obesity (> 35 women, > 25 men)	56.95	33.77	< 0.01
Hypertension (> 140/90 mmHg or treatment)	15.23	24.68	0.00
Total cholesterol ≥ 200 mg/dL	45.03	58.44	0.00
LDL-c ≥ 130 mg/dL	38.41	53.25	< 0.01
Triglycerides ≥ 150 mg/dL	4.64	18.18	< 0.01
Glycaemia 100-125 mg/dL	0.66	27.27	< 0.01
Glycaemia ≥ 126 mg/dL	1.32	3.90	< 0.1
Metabolic syndrome NCEP ATPIII	0.66	18.18	< 0.001
Metabolic syndrome IDF	11.26	10.39	0.42
Metabolic syndrome JIS	11.26	20.78	0.00
Atherogenic dyslipidemia	2.65	3.90	0.26
Lipid triad	2.65	2.60	0.58
Hypertriglyceridemic waist	0.00	2.60	0.01
Atherogenic index total cholesterol/HDL-c moderate-high (> 4.5 women > 5 men)	19.87	15.58	0.10
Atherogenic index triglycerides/HDL-c high (> 3)	3.31	19.48	< 0.01
Atherogenic index LDL-c/HDL-c high (> 3)	19.87	37.66	< 0.01
Atherogenic index total cholesterol-HDL-c high (> 130)	57.62	77.92	< 0.01
SCORE scale moderate-high^*^ (≥ 4)	8.33	18.18	0.01
REGICOR scale moderate-high† (≥ 5)	10.90	14.93	0.01
ERICE scale moderate-high‡ (≥ 10)	2.02	2.99	0.00
DORICA scale moderate-high (≥ 10)	5.3	8.00	< 0.01
Fatty liver index high risk (> 60)	0.93	13.64	< 0.01
BARD scoring system high risk (≥ 2)	20.56	22.73	0.02

* Women n = 176, men n = 192.

† Women n = 248, men n = 268.

‡ Women n = 268, men n = 284.

BMI = body mass index; CUN BAE = Clínica Universitaria de Navarra Body Adiposity Estimator; DORICA = Dyslipidemia Obesity and Cardiovascular Risk in Spain; CORE-BF = Equation Córdoba Estimator Body Fat; ERICE = Spanish Cardiovascular Risk Equation; IDF = International Diabetes Federation; NCEP ATPIII = National Cholesterol Educational Program Adult Treatment Panel III; REGICOR = Registre Gironí del Cor; SCORE = Systematic Coronary Risk Evaluation.

In the multivariate analysis using binary logistic regression, being male, aged 50 years and above, and tobacco consumption were established as covariates. Table 5 shows that those 50 and older have the greatest likelihood of presenting high values for the scales related to CVR.

**Table 5:** Logistic regression analysis

	≥ 50 years vs. < 50 years	Men vs. women	Smokers vs. non smokers
	OR (95%CI)	OR (95%CI)	OR (95%CI)
Waist-to-height ratio > 0.50	2.06 (1.39-3.04)	2.61 (1.78-3.82)	1.66 (1.14-2.42)
BMI obesity	10.59 (6.02-18.63)	0.48 (0.27-0.85)	3.84 (2.19-6.74)
CUN BAE obesity	4.61 (3.15-6.76)	1.59 (1.10-2.29)	NS
ECORE-BF obesity	4.23 (2.89-6.19)	1.78 (1.24-2.55)	NS
Relative fat mass obesity	1.46 (1.01-2.12)	0.46 (0.33-0.65)	NS
Palafolls’s formula obesity	1.85 (1.22-2.80)	2.20 (1.54-3.14)	0.63 (0.44-0.91)
Deurenberg’s formula obesity	3.70 (2.50-5.48)	0.32 (0.22-0.46)	0.40 (0.27-0.58)
Hypertension	2.07 (1.35-3.15)	1.89 (1.25-2.86)	NS
Total cholesterol ≥ 200 mg/dL	10.33 (6.38-16.75)	2.09 (1.46-3.00)	NS
LDL-c ≥ 130 mg/dL	4.76 (3.20-7.07)	2.07 (1.47-2.92)	NS
Triglycerides ≥ 150 mg/dL	5.01 (2.70-9.29)	1.86 (1.10-3.14)	NS
Glycaemia ≥ 126 mg/dL	4.08 (1.35-12.36)	NS	NS
Metabolic syndrome NCEP ATPIII	2.52 (1.42-4.23)	33.20 (8.01-137.54)	NS
Metabolic syndrome IDF	2.86 (1.69-4.85)	2.60 (1.54-4.41)	NS
Metabolic syndrome JIS	2.33 (1.46-3.73)	2.66 (1.65-4.31)	2.95 (1.85-4.68)
Atherogenic dyslipidemia	11.60 (3.81-35.38)	NS	2.68 (1.05-6.80)
Atherogenic index total cholesterol/HDL-c moderate-high	3.75 (2.44-5.78)	NS	NS
Atherogenic index triglycerides/HDL-c high	1.76 (1.02-3.09)	8.64 (4.24-17.62)	2.41 (1.41-4.14)
Atherogenic index LDL-c/HDL-c high	10.83 (6.95-16.88)	3.89 (2.48-6.09)	1.64 (1.08-2.50)
SCORE scale moderate-high	87.21 (35.33-198.21)	NS	4.99 (2.32-10.75)
REGICOR scale moderate-high	1.93 (1.15-3.24)	NS	3.15 (2,55-3.76)
Fatty liver index high risk	4.44 (1.86-10.56)	20.21 (4.57-89.43)	NS
BARD scoring system high risk	12.26 (6.90-21.77)	NS	NS

95%CI = 95% confidence interval; BMI = body mass index; BSI = body surface index; CUN BAE = Clínica Universitaria de Navarra Body Adiposity Estimator; ECORE-BF = Equation COrdoba Estimator Body Fat; IDF = International Diabetes Federation; NCEP ATPIII = National Cholesterol Educational Program Adult Treatment Panel III;

NS = non significance; OR = odds ratio; REGICOR = Registre Gironí del Cor; SCORE = Systematic Coronary Risk Evaluation.

## DISCUSSION

We found that the prevalence of altered values of the different scales that directly or indirectly assess CVR is moderate to high, with men generally suffering the most.

Since we haven’t found any studies in the literature that measure the level of CVR in veterinarians, we will compare our results with those of other health care professionals.

Numerous national studies have assessed the prevalence of arterial hypertension, dyslipidemia, and obesity in health professionals, the first of which focused on medical personnel^36^ and showed, as we did, a high prevalence of these conditions. Another study involving younger health care workers^37^ showed similar results, and a third study,^38^ including different healthcare professionals, such as physicians and dentists, also found similar results. A more recent study,^39^ found the prevalence of obesity in employees of a teaching hospital in Brazil to be 55.5%, similarly to our study.

According to a study involving almost 2000 Mexican health care workers, the determinants of atherogenic indices were male sex, increasing age, increasing waist-to-hip ratio, overweight and obesity, and working as a physician. The occupational category of the physician added risk factors such as stress and adverse psychosocial working conditions, which can potentiate cardiovascular disease.^40^ As in our study, this study showed a high prevalence of atherogenic indices.

In another study^37^ 154 health care workers were found to have moderate and high values on the Framingham scale of 10.3 and 1.3%, respectively, which are in line with our findings. Compared to our study, this study had higher prevalence of hypertension (33%), overweight and obesity (66%), and dyslipidemia (33%).

Among the strengths of the study are the large sample size, more than 700 veterinarians, the wide variety of scales used, including 14 scales that assess overweight and obesity, 6 for fatty liver, 6 for CVR, 4 atherogenic indices, and 3 for metabolic syndrome, and to our knowledge, it is the first study that addresses the level of CVR in veterinarians, which could make it a reference for subsequent studies in this group.

The most important limitation is that it was conducted in a specific geographical area, which makes it difficult to extrapolate the results to other countries.

## CONCLUSIONS

The prevalence of scales related to CVR in veterinarians can be considered moderate-high, especially if we take into account that the average age of these workers is not very high.
